# Supplementary material for: Energy-temperature uncertainty relation in quantum thermodynamics
Source: Nat Commun. 2018 Jun 6;9:2203. doi: 10.1038/s41467-018-04536-7 (PMC5989247; doi:10.1038/s41467-018-04536-7)
Supplement: Supplementary file 1 — Supplementary Information [file 41467_2018_4536_MOESM1_ESM.pdf]

# – Supplementary Information –

Energy-temperature uncertainty relation in quantum thermodynamics

H. J. D. Miller* and J. Anders†
Department of Physics and Astronomy, University of Exeter, Stocker Road, EX4 4QL, United Kingdom.
(Dated: April 23, 2018)

SUPPLEMENTARY NOTE 1: DERIVATION OF UPPER BOUND ON THE QUANTUM FISHER INFORMATION

We begin by considering an exponential state $\hat{\rho}_\theta$ dependent on smooth parameter θ of the form $\hat{\rho}_\theta = e^{-\hat{A}_\theta}/Z_\theta$, where $Z_\theta = \mathrm{tr}[e^{-\hat{A}_\theta}]$ and $\hat{A}_\theta$ is some positive hermitian operator. Suppressing the dependence on θ for now, let us denote the spectral decomposition by $\hat{\rho}_\theta = \sum_n p_n |\psi_n\rangle\langle\psi_n|$ where the eigenstates satisfy $\hat{A}_\theta|\psi_n\rangle = \lambda_n|\psi_n\rangle$. We arrange the sum in decreasing order, so that $p_n \geq p_m$ if $n < m$. The quantum Fisher information (QFI) with respect to θ is then $F(\theta) := \mathrm{tr}[\hat{\rho}_\theta\, \hat{L}_\theta^2]$, where $\hat{L}_\theta$ is the operator satisfying

$$\partial_\theta \hat{\rho}_\theta = \frac{1}{2}\{\hat{\rho}_\theta, \hat{L}_\theta\}. \tag{1}$$

By expanding both sides of (1) in the basis of $\hat{\rho}_\theta$, one may show that the QFI can be written as follows [1]:

$$F(\theta) = 2\sum_{n,m} \frac{|\langle\psi_n|\partial_\theta\hat{\rho}_\theta|\psi_m\rangle|^2}{p_n + p_m}. \tag{2}$$

We define the operator $\hat{B}_\theta = \partial_\theta \hat{A}_\theta$ and note that $\mathsf{Var}[\hat{\rho}_\theta, \hat{B}_\theta] = \mathrm{tr}[(\delta\hat{B}_\theta)^2\, \hat{\rho}_\theta]$, where $\delta\hat{B}_\theta = \hat{B}_\theta - \mathrm{tr}[\hat{B}_\theta\, \hat{\rho}_\theta]$ and $\mathrm{tr}[\hat{B}_\theta\, \hat{\rho}_\theta] = -\partial_\theta \ln Z_\theta$. Given the exponential form of $\hat{\rho}_\theta$, we use the following integral expression to expand the derivative [2]:

$$\partial_\theta[e^{-\hat{A}_\theta}] := -\int_0^1 da\; e^{-(1-a)\hat{A}_\theta}\; \partial_\theta[\hat{A}_\theta]\; e^{-a\hat{A}_\theta}, \tag{3}$$

where $a \in \mathbb{R}$ is a real number. Using this the QFI becomes

$$\begin{aligned}
F(\theta) &= 2\sum_{n,m} \frac{|\langle\psi_n|\partial_\theta e^{-(\hat{A}_\theta + \ln Z_\theta)}|\psi_m\rangle|^2}{p_n + p_m}, \\
&= \frac{2}{Z_\theta^2}\sum_{n,m} \frac{1}{p_n + p_m}\left|\langle\psi_n| \int_0^1 da\; e^{-(1-a)\hat{A}_\theta}\delta\hat{B}_\theta\; e^{-a\hat{A}_\theta}|\psi_m\rangle\right|^2, \\
&= \sum_n p_n \big|\langle\psi_n|\delta\hat{B}_\theta|\psi_n\rangle\big|^2 + \frac{4}{Z^2}\sum_{n<m}\frac{1}{p_n+p_m}\big|\langle\psi_m|\hat{B}_\theta|\psi_n\rangle\big|^2 \left[\int_0^1 da\; e^{-\left(a\lambda_m + (1-a)\lambda_n\right)}\right]^2, \\
&= \sum_n p_n \big|\langle\psi_n|\delta\hat{B}_\theta|\psi_n\rangle\big|^2 + 4\sum_{n<m}\frac{(p_n-p_m)^2}{p_n+p_m}\big|\langle\psi_n|\hat{B}_\theta|\psi_m\rangle\big|^2 \frac{1}{(\ln p_n - \ln p_m)^2}.
\end{aligned} \tag{4}$$

Let us now use the following expression for the variance:

$$\begin{aligned}
\mathsf{Var}[\hat{\rho}_\theta, \hat{B}_\theta] &= \sum_{n,m}\frac{p_n+p_m}{2}\big|\langle\psi_n|\delta\hat{B}_\theta|\psi_m\rangle\big|^2, \\
&= \sum_n p_n \big|\langle\psi_n|\delta\hat{B}_\theta|\psi_n\rangle\big|^2 + \sum_{n\neq m}\frac{p_n+p_m}{2}\big|\langle\psi_n|\delta\hat{B}_\theta|\psi_m\rangle\big|^2, \\
&= \sum_n p_n \big|\langle\psi_n|\delta\hat{B}_\theta|\psi_n\rangle\big|^2 + \sum_{n<m}(p_n+p_m)\big|\langle\psi_n|\delta\hat{B}_\theta|\psi_m\rangle\big|^2.
\end{aligned} \tag{5}$$

---

* hm419@exeter.ac.uk
† janet@qipc.org

Following this we now substitute (5) into (4), which gives the following expression for the QFI:

$$F(\theta) = \mathsf{Var}[\hat{\rho}_\theta, \hat{B}_\theta] + \sum_{n<m} \left[\left(\frac{2(p_n - p_m)}{\ln(p_n/p_m)} \right) \left(\frac{2(p_n - p_m)}{(p_n + p_m)\ln(p_n/p_m)} \right) - (p_n + p_m) \right] \left| \langle \psi_n | \hat{B}_\theta | \psi_m \rangle \right|^2. \tag{6}$$

We now turn to the average WYD skew information of observable $\hat{B}_\theta$, which is given by

$$Q[\hat{\rho}_\theta, \hat{B}_\theta] = -\frac{1}{2} \int_0^1 da \; \mathrm{tr}\big[[\hat{B}_\theta, \hat{\rho}_\theta^a][\hat{B}_\theta, \hat{\rho}_\theta^{1-a}] \big]. \tag{7}$$

It follows from the analysis of [3] that for a full-rank state $Q[\hat{\rho}_\theta, \hat{B}_\theta]$ can also be expanded in the eigenbasis of $\hat{\rho}_\theta$, yielding

$$Q[\hat{\rho}_\theta, \hat{B}_\theta] = \sum_{n<m} \left(p_n + p_m - \frac{2(p_n - p_m)}{\ln p_n - \ln p_m} \right) \left| \langle \psi_n | \hat{B}_\theta | \psi_m \rangle \right|^2. \tag{8}$$

We now bound the QFI following on from (6):

$$\begin{aligned} F(\theta) &= \mathsf{Var}[\hat{\rho}_\theta, \hat{B}_\theta] + \sum_{n<m} \left[\left(\frac{2(p_n - p_m)}{\ln(p_n/p_m)} \right) \left(\frac{2(p_n - p_m)}{(p_n + p_m)\ln(p_n/p_m)} \right) - (p_n + p_m) \right] \left| \langle \psi_n | \hat{B}_\theta | \psi_m \rangle \right|^2, \\ &\leq \mathsf{Var}[\hat{\rho}_\theta, \hat{B}_\theta] + \sum_{n<m} \left[\frac{2(p_n - p_m)}{\ln(p_n/p_m)} - (p_n + p_m) \right] \left| \langle \psi_n | \hat{B}_\theta | \psi_m \rangle \right|^2, \\ &= \mathsf{Var}[\hat{\rho}_\theta, \hat{B}_\theta] - Q[\hat{\rho}_\theta, \hat{B}_\theta], \end{aligned} \tag{9}$$

where in the second line we used the fact that $(p_n - p_m)/\ln(p_n/p_m) \geq 0$ since $p_n \geq p_m$ for $n < m$, and the inequality

$$\frac{x-1}{x+1} \leq \ln \sqrt{x}; \qquad x \geq 1, \tag{10}$$

identifying $x = p_n/p_m \geq 1$. This allowed us to use

$$\left(\frac{2(p_n - p_m)}{(p_n + p_m)\ln(p_n/p_m)} \right) \leq 1, \tag{11}$$

for each term inside the sum. In the third line we used the expression (8) for the skew information. Finally we recall that $\mathsf{Var}[\hat{\rho}_\theta, \hat{B}_\theta] - Q[\hat{\rho}_\theta, \hat{B}_\theta] = K[\hat{\rho}_\theta, \hat{B}_\theta]$, and so we arrive at the bound on the QFI stated in the main text:

$$F(\theta) \leq K[\hat{\rho}_\theta, \hat{B}_\theta]. \tag{12}$$

SUPPLEMENTARY NOTE 2: DERIVATION OF THE MODIFIED FLUCTUATION-DISSIPATION RELATION

Denote the operator $\delta\hat{E}^*_{\mathcal{S}} := \hat{E}^*_{\mathcal{S}}(T) - \langle \hat{E}^*_{\mathcal{S}}(T) \rangle$ as the deviation in internal energy and $\Delta U^2_{\mathcal{S}} = \mathsf{tr}[\hat{\pi}_{\mathcal{S}}(T)(\delta\hat{E}^*_{\mathcal{S}})^2]$ as the energy variance, dropping the temperature dependence for now. We now evaluate the average WYD skew information of the internal energy:

$$\begin{aligned} Q[\hat{\pi}_{\mathcal{S}}, \hat{E}^*_{\mathcal{S}}] &= \Delta U^2_{\mathcal{S}} - K[\hat{\pi}_{\mathcal{S}}, \hat{E}^*_{\mathcal{S}}], \\ &= \Delta U^2_{\mathcal{S}} - \int_0^1 da \; \mathsf{tr}[\hat{\pi}_{\mathcal{S}}^{1-a} \delta\hat{E}^*_{\mathcal{S}} \; \hat{\pi}^a_{\mathcal{S}} \; \delta\hat{E}^*_{\mathcal{S}}], \\ &= \Delta U^2_{\mathcal{S}} - \int_0^1 da \; \mathsf{tr}[e^{-(1-a)(\beta\hat{H}^*_{\mathcal{S}} + \ln Z^*_{\mathcal{S}})} \delta\hat{E}^*_{\mathcal{S}} e^{-a(\beta\hat{H}^*_{\mathcal{S}} + \ln Z^*_{\mathcal{S}})} \delta\hat{E}^*_{\mathcal{S}}], \\ &= \Delta U^2_{\mathcal{S}} + \mathsf{tr}[\delta\hat{E}^*_{\mathcal{S}} \; \partial_\beta \hat{\pi}_{\mathcal{S}}], \\ &= \Delta U^2_{\mathcal{S}} + \mathsf{tr}[\hat{E}^*_{\mathcal{S}} \; \partial_\beta \hat{\pi}_{\mathcal{S}}], \\ &= \Delta U^2_{\mathcal{S}} - T^2 \mathsf{tr}[\hat{E}^*_{\mathcal{S}} \; \partial_T \hat{\pi}_{\mathcal{S}}], \\ &= \Delta U^2_{\mathcal{S}} - T^2 \partial_T \mathsf{tr}[\hat{E}^*_{\mathcal{S}} \; \hat{\pi}_{\mathcal{S}}] + T^2 \mathsf{tr}[\partial_T \hat{E}^*_{\mathcal{S}} \; \hat{\pi}_{\mathcal{S}}], \\ &= \Delta U^2_{\mathcal{S}} - T^2 C_{\mathcal{S}}(T) + T^2 \langle \partial_T \hat{E}^*_{\mathcal{S}} \rangle, \end{aligned} \tag{13}$$

where we used the relation $\delta \hat{E}_S^* = \partial_\beta(\beta \hat{H}_S^* + \ln Z_S^*)$ and (3) in the fourth line, and the fact that the operator $\partial_\beta \hat{\pi}_S$ is traceless in the fifth line. Rearranging both sides yields the modified fluctuation-dissipation relation:

$$C_S(T) = \frac{\Delta U_S^2}{T^2} - \frac{Q[\hat{\pi}_S, \hat{E}_S^*]}{T^2} + \langle \partial_T \hat{E}_S^* \rangle. \tag{14}$$

SUPPLEMENTARY NOTE 3: CALCULATIONS FOR THE DAMPED HARMONIC OSCILLATOR

As stated in the main text the mean force Hamiltonian of the probe is given by $\hat{H}_S^*(T) = \omega_T(\hat{n}_T + \frac{1}{2})$, with $\hat{n}_T = \hat{a}_T^\dagger \hat{a}_T$ and $\hat{a}_T = \sqrt{\frac{A_T}{2}}(\hat{x} + i\frac{\hat{p}}{A_T})$. We will set $\hbar = 1$ throughout and A_T is given by $A_T = \sqrt{\langle \hat{p}^2 \rangle / \langle \hat{x}^2 \rangle}$, where the effective mass and frequency are given respectively by

$$M_T = \omega_T^{-1} \sqrt{\frac{\langle \hat{p}^2 \rangle}{\langle \hat{x}^2 \rangle}}, \tag{15}$$

$$\omega_T = 2T \,\text{arcoth}(2\sqrt{\langle \hat{p}^2 \rangle \langle \hat{x}^2 \rangle}). \tag{16}$$

We can diagonalise the state of the probe in terms of the number states of $\hat{n}_T$, so $\hat{\pi}_S(T) = e^{-\beta \hat{H}_S^*(T)}/Z_S^* = \sum_{n=0}^\infty p_n |n\rangle\langle n|$ where

$$p_n = \frac{e^{-\beta \epsilon_n}}{Z_S^*}, \qquad Z_S^* = 2\,\text{sinh}^{-1}\left(\frac{\beta \omega_T}{2}\right), \tag{17}$$

and $\epsilon_n = \omega_T(n + \frac{1}{2})$. Furthermore, from the main text the internal energy operator is given by

$$\hat{E}_S^*(T) = \alpha_T \hat{H}_S^*(T) - g_T \frac{\hat{a}_T^2 + (\hat{a}_T^\dagger)^2}{2}, \tag{18}$$

with $\alpha_T = 1 - \frac{\omega_T'}{\omega_T} T$ and $g_T = \omega_T T \frac{A_T'}{A_T}$. The functions g_T and α_T are determined by the effective mass and frequency of the oscillator defined above, so we need to calculate $\langle \hat{x}^2 \rangle$ and $\langle \hat{p}^2 \rangle$. In the continuum limit $N \to \infty$ the exact expressions for the quadratures are found to be [4]:

$$\langle \hat{x}^2 \rangle = \frac{1}{M\beta\omega^2} + \frac{\hbar}{M\pi} \sum_{i=1}^3 \left[\frac{(\lambda_i - \omega_\text{D})\Gamma(1 + \frac{\beta\hbar\lambda_i}{2\pi})}{(\lambda_{i+1} - \lambda_i)(\lambda_{i-1} - \lambda_i)} \right], \tag{19}$$

$$\langle \hat{p}^2 \rangle = M\omega^2 \langle \hat{x}^2 \rangle + \frac{\hbar M \gamma \omega_\text{D}}{\pi} \sum_{i=1}^3 \left[\frac{\lambda_i \Gamma(1 + \frac{\beta\hbar\lambda_i}{2\pi})}{(\lambda_{i+1} - \lambda_i)(\lambda_{i-1} - \lambda_i)} \right], \tag{20}$$

where $\Gamma(z)$ is the digamma function and λ_i are the characteristic frequencies of the oscillator. In the limit of a large cutoff frequency, $\omega_\text{D} \gg \omega, \gamma$ the frequencies are given by

$$\lambda_1 = \frac{\gamma}{2} + \sqrt{\frac{\gamma^2}{4} - \omega^2}, \tag{21}$$

$$\lambda_2 = \frac{\gamma}{2} - \sqrt{\frac{\gamma^2}{4} - \omega^2}, \tag{22}$$

$$\lambda_3 = \omega_D - \gamma. \tag{23}$$

Due to the complicated dependence on T we will not present the exact analytic expressions for g_T and α_T, but we will proceed to calculate $C_S(T)$, $F_S(T)$ and $\langle \partial_T \hat{E}_S^* \rangle$. The average internal energy is found to be

$$\langle \hat{E}_S^* \rangle = \alpha_T \langle \hat{H}_S^*(T) \rangle, \tag{24}$$

$$\langle \hat{H}_S^*(T) \rangle = \frac{\omega_T}{2} \text{coth}\left(\frac{\beta\omega_T}{2}\right). \tag{25}$$

The heat capacity can now be calculated by differentiating the average energy:

$$
\begin{aligned}
C_S(T) &= \partial_T \langle \hat{E}_S^* \rangle, \\
&= \alpha'_T \frac{\omega_T}{2} \coth\left(\frac{\beta\omega_T}{2}\right) + \alpha_T \frac{\omega'_T}{2} \coth\left(\frac{\beta\omega_T}{2}\right) - \frac{\alpha_T\omega_T(\omega'_T\beta - \omega_T\beta^2)}{4\sinh^2\left(\frac{\beta\omega_T}{2}\right)}, \\
&= \frac{1}{2}\coth\left(\frac{\beta\omega_T}{2}\right)(\alpha'_T\omega_T + \alpha_T\omega'_T) - \frac{\alpha_T\omega_T\beta(\omega'_T - \omega_T\beta)}{4\sinh^2\left(\frac{\beta\omega_T}{2}\right)}.
\end{aligned} \tag{26}
$$

In order to calculate the QFI (2) and skew information (8) we will need to obtain the elements $E_{nm} = |\langle n|\delta\hat{E}^*|m\rangle|^2$. Firstly one finds the following:

$$
\begin{aligned}
\langle n|\delta\hat{E}^*|m\rangle &= \langle n|\hat{E}_S^*|m\rangle - \langle \hat{E}_S^*\rangle \delta_{n,m}, \\
&= \alpha_T \langle n|\hat{H}_S^*(T)|m\rangle - g_T \langle n|\frac{\hat{a}_T^2 + (\hat{a}_T^\dagger)^2}{2}|m\rangle - \langle \hat{E}_S^*\rangle \delta_{n,m}, \\
&= \alpha_T(\epsilon_n - \langle \hat{H}_S^*(T)\rangle)\delta_{n,m} - g_T\left(\frac{\sqrt{m}\sqrt{m-1}}{2}\delta_{n,m-2} + \frac{\sqrt{n}\sqrt{n-1}}{2}\delta_{m,n-2}\right),
\end{aligned} \tag{27}
$$

where $\delta_{n,m}$ represents the Kronecker-Delta function. Squaring both sides yields

$$
E_{nm} = \alpha_T^2(\epsilon_n - \langle \hat{H}_S^*(T)\rangle)^2\delta_{n,m} + \frac{g_T^2}{4}\Big((n+2)(n+1)\delta_{n+2,m} + (m+2)(m+1)\delta_{m+2,n}\Big), \tag{28}
$$

The variance in internal energy is given by (5), so that

$$
\begin{aligned}
\text{Var}[\hat{\pi}_S, \hat{E}_S^*] &= \sum_{n,m=0}^{\infty} p_n E_{nm}, \\
&= \alpha_T^2 \text{Var}[\hat{\pi}_S, \hat{H}_S^*] + \frac{g_T^2}{2}\sum_{n=0}^{\infty} p_n(n+1)(n+2), \\
&= \alpha_T^2 \text{Var}[\hat{\pi}_S, \hat{H}_S^*] + g_T^2 \sinh\left(\frac{\beta\omega_T}{2}\right)\sum_{n=0}^{\infty}(n+1)(n+2)e^{-\beta\omega_T(n+\frac{1}{2})} \\
&= \alpha_T^2 \text{Var}[\hat{\pi}_S, \hat{H}_S^*] + 2g_T^2 \sinh\left(\frac{\beta\omega_T}{2}\right)\left(\frac{e^{-\frac{\beta\omega_T}{2}}}{(1-e^{-\beta\omega_T})^3}\right), \\
&= \frac{\alpha_T^2\omega_T^2}{4\sinh^2\left(\frac{\beta\omega_T}{2}\right)} + 2g_T^2 \sinh\left(\frac{\beta\omega_T}{2}\right)\left(\frac{e^{-\frac{\beta\omega_T}{2}}}{(1-e^{-\beta\omega_T})^3}\right),
\end{aligned} \tag{29}
$$

where we used the series $\sum_{n=0}^{\infty}(n+1)(n+2)x^n = 2/(1-x)^3$ for $|x| < 1$ and that the variance of $\hat{H}_S^*(T)$ is

$$
\text{Var}[\hat{\pi}_S, \hat{H}_S^*] = \frac{\omega_T^2}{4\sinh^2\left(\frac{\beta\omega_T}{2}\right)}. \tag{30}
$$

We now compute the QFI, using (2) and the fact that $p_n \pm p_{n+2} = p_n(1 \pm e^{-2\beta\omega_T})$:

$$
\begin{aligned}
T^4 F_S(T) &= \sum_{n=0}^{\infty} p_n E_{nn} + 4\sum_{n<m} \frac{(p_n - p_m)^2}{(p_n + p_m)\ln^2\left(\frac{p_n}{p_m}\right)} E_{nm}, \\
&= \alpha_T^2 \text{Var}[\hat{\pi}_S, \hat{H}_S^*] + \frac{g_T^2}{\beta^2\omega_T^2}\sum_{n=0}^{\infty}\frac{(p_n - p_{n+2})^2}{p_n + p_{n+2}}(n+2)(n+1), \\
&= \alpha_T^2 \text{Var}[\hat{\pi}_S, \hat{H}_S^*] + \frac{g_T^2(1-e^{-2\beta\omega_T})^2}{\beta^2\omega_T^2(1+e^{-2\beta\omega_T})}\sum_{n=0}^{\infty} p_n(n+2)(n+1), \\
&= \alpha_T^2 \text{Var}[\hat{\pi}_S, \hat{H}_S^*] + \frac{g_T^2}{\beta^2\omega_T^2}\sinh\left(\frac{\beta\omega_T}{2}\right)\left(\frac{(1-e^{-2\beta\omega_T})^2}{(1+e^{-2\beta\omega_T})}\right)\left(\frac{e^{-\frac{\beta\omega_T}{2}}}{(1-e^{-\beta\omega_T})^3}\right), \\
&= \frac{\alpha_T^2\omega_T^2}{4\sinh^2\left(\frac{\beta\omega_T}{2}\right)} + \frac{g_T^2}{\beta^2\omega_T^2}\sinh\left(\frac{\beta\omega_T}{2}\right)\left(\frac{(1-e^{-2\beta\omega_T})^2}{(1+e^{-2\beta\omega_T})}\right)\left(\frac{e^{-\frac{\beta\omega_T}{2}}}{(1-e^{-\beta\omega_T})^3}\right),
\end{aligned} \tag{31}
$$

We also require the average skew information, which can be obtained using (8):

$$\begin{aligned} Q[\hat{\pi}_S, \hat{E}_S^*] &= \sum_{n<m} \left(p_n + p_m - \frac{2(p_n - p_m)}{\ln p_n - \ln p_m} \right) E_{nm}, \\ &= \frac{g_T^2}{4} \sum_{n=0}^{\infty} \left(p_n + p_{n+2} - \frac{2(p_n - p_{n+2})}{\ln p_n - \ln p_{n+2}} \right) (n+2)(n+1), \\ &= g_T^2 \, \mathsf{sinh}\left(\frac{\beta\omega_T}{2} \right) \frac{e^{-\frac{\beta\omega_T}{2}}}{(1 - e^{-\beta\omega_T})^3} \left(1 + e^{-2\beta\omega_T} - \frac{T}{\omega_T}(1 - e^{-2\beta\omega_T}) \right). \end{aligned} \tag{32}$$

To calculate the term $\langle \partial_T \hat{E}^* \rangle$ we use the fluctuation-dissipation relation (14):

$$\begin{aligned} \langle \partial_T \hat{E}_S^* \rangle &= C_S(T) + \frac{Q[\hat{\pi}_S, \hat{E}_S^*] - \mathsf{Var}[\hat{\pi}_S, \hat{E}_S^*]}{T^2}, \\ &= C_S(T) - \frac{g_T^2}{T\omega_T} \mathsf{sinh}\left(\frac{\beta\omega_T}{2} \right) \left(\frac{(1 - e^{-2\beta\omega_T})}{(1 - e^{-\beta\omega_T})^3} \right) e^{-\frac{\beta\omega_T}{2}}. \end{aligned} \tag{33}$$

[1] Hayashi, M. *Quantum Information Theory: Mathematical Foundation* (Springer, Berlin, 2017).

[2] Wilcox, R. M. Exponential operators and parameter differentiation in quantum physics. *J. Math. Phys.* **8**, 962–982 (1967).

[3] Li, X., Li, D., Huang, H., Li, X. & Kwek, L. C. Averaged Wigner-Yanase-Dyson information as a quantum uncertainty measure. *Euro. Phys J. D* **64**, 147–153 (2011).

[4] Grabert, H., Weiss, U. & Talkner, P. Quantum theory of the damped harmonic oscillator. *Z. Phys. B.* **55**, 87–94 (1984).
